# Urethral Steinstrasse following Laser Lithotripsy of Prostatic Urethral Calculi

**DOI:** 10.1155/2018/3459347

**Published:** 2018-05-09

**Authors:** Jennifer Den, Preston S. Kerr, Tamer J. Dafashy, Christopher D. Kosarek, Robyn L. Roberts, J. Nicholas Sreshta

**Affiliations:** Division of Urology, Department of Surgery, University of Texas Medical Branch, Galveston, TX, USA

## Abstract

Symptomatic prostatic calculi are rare occurrences with several management options, the most popular of which is currently transurethral laser lithotripsy. This is a generally well-tolerated procedure with minimal complications. To date, no reported episodes of steinstrasse at the urethral level following prostatic calculi lithotripsy have been documented to our knowledge. We report a unique case of acute urinary retention secondary to obstructive calculi fragments following a transurethral laser lithotripsy of large prostatic calculi, further complicated by stricture at the fossa navicularis.

## 1. Introduction

Symptomatic and obstructive calculi of the prostatic urethra are an uncommon clinical entity. They are usually seen in older men and are typically incidental findings on digital rectal examination, CT, or other forms of radiological investigation [1]. The exact prevalence of prostatic calculi is not known, with reports suggesting a range of 7% to 70%. Prostatic calculi may be found in association with normal glands, benign prostatic hyperplasia, chronic bacterial prostatitis, or prostate adenocarcinoma and may vary in number from a single calculus to several hundred [2]. Traditionally, prostatic calculi were managed by open surgery [3]; however, transurethral laser lithotripsy has been shown to be a less invasive and more effective method [1, 4]. Steinstrasse, or “street of stones,” is a known, albeit uncommon, complication of extracorporeal shock wave lithotripsy (ESWL) when incomplete fragmentation occurs, leaving behind residual stone fragments that obstruct urinary flow [5]. The literature has yet to document this occurring following laser lithotripsy of large prostatic calculi.

Due to the rarity of large recurrent symptomatic prostatic calculi and the occurrence of urethral steinstrasse after laser lithotripsy, we chose to report this case, which occurred over the course of six months.

## 2. Case Presentation

**Figure 1:**
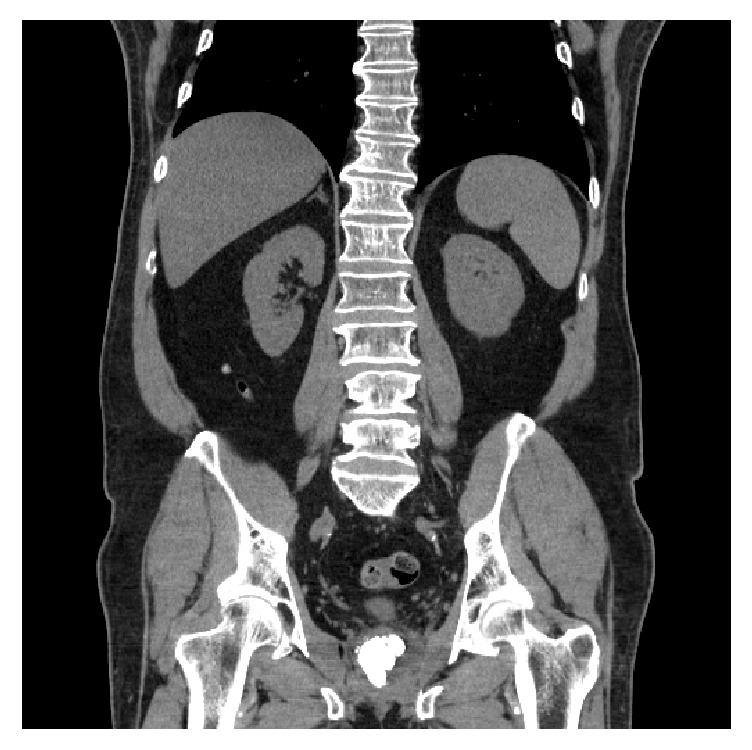
Coronal view of CT abdomen and pelvis noting a large burden of prostate stones.

**Figure 2:**
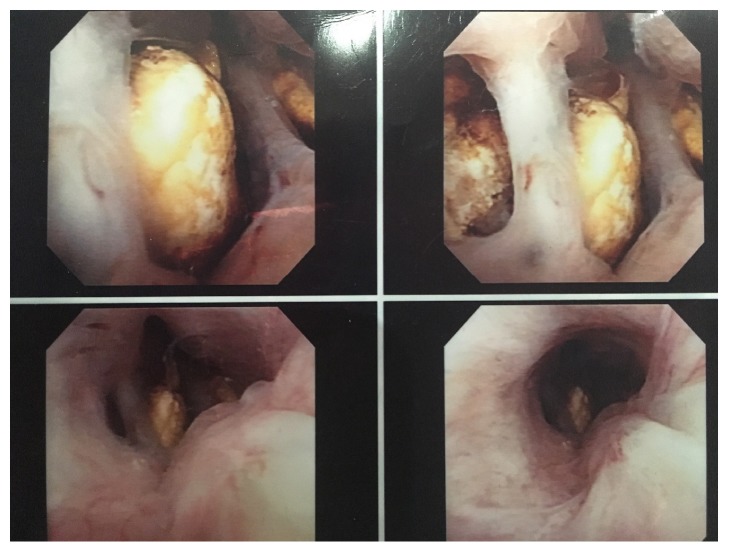
Axial view of CT abdomen and pelvis noting a large burden of prostate stones.

**Figure 3:**
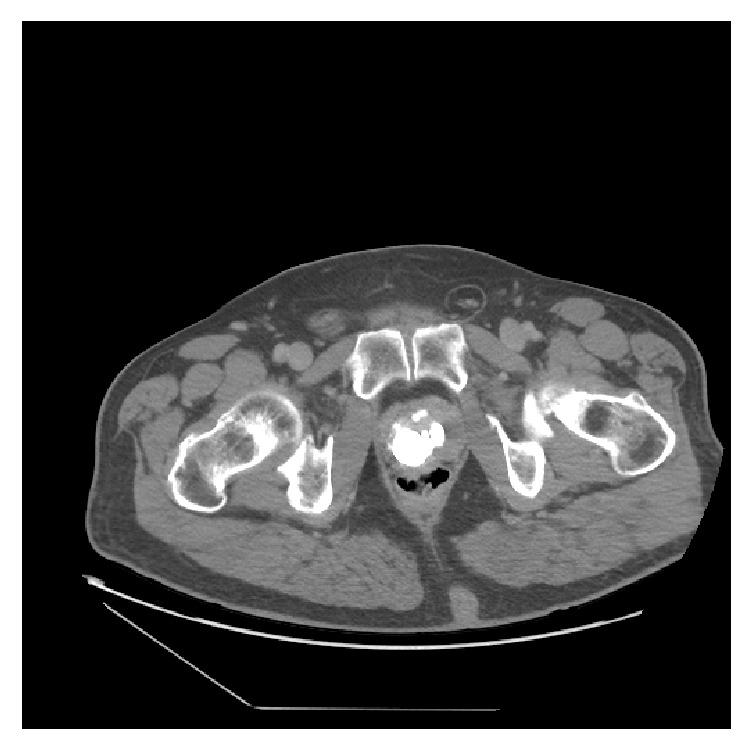
Cystoscopic view from the verumontanum noting a large burden of prostate stones.

A 57-year-old male with a history of coronary artery disease and type 2 diabetes mellitus presented to the clinic after an isolated episode of gross hematuria and progressively worsening lower urinary tract symptoms (LUTS). He endorsed a history of prostatic calculi requiring surgical intervention as a teenager. His digital rectal examination demonstrated a diffusely firm prostate with an irregular contour. Prostate specific antigen was found to be 0.56 ng/mL and he had no known family history of prostate cancer. His postvoid residual (PVR) measured 13 mL. The remainder of his physical examination was unremarkable. A CT urogram was remarkable for only an enlarged prostate and several prostatic calculi, with the largest measuring 2.4 cm × 1.6 cm (Figures 1 and 2). Cystoscopy in the clinic showed several large obstructive prostatic calculi that would not allow passage of the scope into the bladder (Figure 3).

The patient subsequently underwent a transurethral resection of the prostate (TURP) and holmium laser (200-micron fiber, 15–50 Hz, 0.2–0.8 J) lithotripsy of multiple prostatic calculi. The fragments were removed via a cystoscopic grasper and a Boston Scientific 1.9 French Zero-Tip basket. He was discharged with an indwelling catheter. Shortly after removal of the indwelling catheter, the patient experienced difficulty voiding and was found to be in urinary retention. Office cystoscopy revealed several fragmented stones at the level of the prostatic urethra, similar to steinstrasse. An indwelling catheter was placed to relieve obstruction and the patient underwent repeat cystoscopy under anesthesia with removal of the remaining fragments.

At 3-month follow-up, the patient returned to the clinic with restricted flow, a spraying stream, and a PVR of 87 mL. Office cystoscopy demonstrated an 8–10-French stricture in the fossa navicularis requiring a urethral meatotomy. Subsequent cystoscopy a month later demonstrated a normal urethra and a singular calculus in the prostatic fossa that was noted to be nonobstructive. Two weeks later at follow-up, the patient reported he was urinating well.

## 3. Discussion

Prostatic calculi are thought to form during inflammatory conditions when prostatic secretions precipitate and the corpora amylacea calcify [2]. They can be classified based on their origin: endogenous (formed de novo in the urethra) and exogenous (formed in the upper urinary tract with secondary downward descent) [6]. Endogenous calculi are smaller and formed directly from precipitation of elements in prostatic secretions [2, 7]. These do not commonly cause acute symptoms due to their slow development [2]. Exogenous calculi are formed from constituents of urine and are most often composed of calcium oxalate and phosphate [2, 8]. They are more common and are known to cause acute symptoms such as urinary retention, frequency, weak stream, and/or dysuria. They are often associated with urethral stricture diseases or other forms of urethral obstruction [6, 8]. Exogenous prostatic calculi are thought to originate as stones more proximal in the renal system that become lodged in the prostatic urethra [3]. Due to the size and the prior history of a prostate stone, this patient most likely had endogenous stones.

Prostatic calculi usually present with a normal clinical examination and are typically discovered incidentally. Stones generally range between 0.5 mm and 5 mm, although there have been bigger stones described [7]. In this case, the patient's diagnosis of prostate calculi, which measured 2.4 cm × 1.6 cm, was made with CT and cystourethroscopy.

Treatment and management of calculi are contingent on the condition of the urethra and the size, shape, and position of the calculus. Open surgery was previously the most common approach [3]. Given the advancement of endoscopic surgery, less morbid methods are currently employed. Methods of treatment include transposing the calculus back into the bladder if it is located in the posterior urethra, or performing meatotomy for stones located in the fossa navicularis or external meatus [8]. Review of the literature indicates that transurethral holmium-YAG laser lithotripsy offers an attractive approach, specifically for symptomatic prostatic calculi. It is relatively safe without long-term complications [1, 4]. This operation pulverizes stones and eliminates fragments until they can pass spontaneously into the bladder.

Steinstrasse is a rare complication most commonly described after ESWL for nephrolithiasis. Fragmentation after lithotripsy most commonly occurs in the distal ureter due to the narrowing at the ureterovesicular junction [9]. In this case, however, we use steinstrasse to describe a prostatic urethral obstruction.

Urethral steinstrasse is uncommon because fragments that pass through the ureterovesical junction can often traverse a much larger caliber [10]. The average urethral caliber in an adult male that is compatible with normal micturition is 6 mm or greater [11]. In our reported case, laser lithotripsy was used to resect and fragment the patient's prostatic calculi and urethral meatotomy was used to manage fossa navicularis stricture. Urethral steinstrasse developed following laser lithotripsy, which makes this case unique.

In this instance, the patient underwent a cystourethroscopy, although the indwelling catheter may have helped pass some of the residual stones. Steinstrasse in very rare cases can spontaneously resolve itself [9].

Patients require long-term surveillance to avoid recurrent stone formation, which can include metabolic evaluation or treatment of predisposing factors such as urethral stenosis or diverticula [12]. Recurrence of prostatic calculi may be observed for reasons such as reformation of de novo calculi in prostate gland cavities, formation of new calculi, or incomplete excision of related diverticula [1]. Patients with predisposing conditions such as urethral stricture or neurogenic bladder are especially prone to recurrence due to repeat urinary tract infections, stagnation, or obstruction [1]. For this particular case, urethral steinstrasse may have been prevented had the patient's indwelling catheter remained in place until stone fragments were no longer visible. The lack of a standardized surveillance and treatment protocol poses many questions that future research needs to address.

## References

[1] Goyal N. K., Goel A., Sankhwar S. (2013). Transurethral holmium-YAG laser lithotripsy for large symptomatic prostatic calculi: Initial experience.

[2] Venyo A. Prostatic calculi: a review of the literature. http://www.webmedcentral.com.

[3] McDonald H. P., Upchurch W. E., Sturdevant C. E. (1955). Treatment of prostatic calculi.

[4] Walker B. R., Hamilton B. D. (2001). Urethral calculi managed with transurethral Holmium laser ablation..

[5] Daddessi A., Vittori M., Racioppi M., Pinto F., Sacco E., Bassi P. (2012). Complications of extracorporeal shock wave lithotripsy for urinary stones: To know and to manage them-a review.

[6] Prabhuswamy V. K., Tiwari R., Krishnamoorthy R. (2013). A giant dumbbell shaped vesico-prostatic urethral calculus: a case report and review of literature.

[7] Najoui M., Qarro A., Ammani A., Alami M. (2013). Giant prostatic calculi.

[8] Kotkar K., Thakkar R., Songra M. (2011). Giant urethral calculus.

[9] Sayed M. A.-B., El-Taher A. M., Aboul-Ella H. A., Shaker S. E. (2001). Steinstrasse after extracorporeal shockwave lithotripsy: Aetiology, prevention and management.

[10] Brahmbhatt Y. G., Schulsinger D. A., Wadhwa N. K. (2009). Urethral steinstrasse in renal transplantation.

[11] Manoliu R. A. (1982). Urethral calibre measurements on micturition cystourethrograms in adult males. II. Subvesical obstruction.

[12] Naouar S., Khalifa B. B., Braiek S., Kamel R. E. (2016). Recurrent Giant Prostatic Urethral Calculus: A Case Report and Mini-Review of the Literature.

